# Analysis of different HER‐2 mutations in breast cancer progression and drug resistance

**DOI:** 10.1111/jcmm.12662

**Published:** 2015-08-25

**Authors:** Zijia Sun, Yaqin Shi, Yan Shen, Lulu Cao, Wenwen Zhang, Xiaoxiang Guan

**Affiliations:** ^1^ Department of Medical OncologyJinling HospitalMedical School of Nanjing UniversityNanjingChina; ^2^ Department of Medical OncologyJinling HospitalSchool of MedicineSouthern Medical UniversityGuangzhouChina

**Keywords:** breast cancer, HER‐2, HER‐2 mutation, variants, cancer risk, resistance

## Abstract

Studies over the last two decades have identified that amplified human epidermal growth factor receptor (HER‐2; c‐erbB‐2, neu) and its overexpression have been frequently implicated in the carcinogenesis and prognosis in a variety of solid tumours, especially breast cancer. Lots of painstaking efforts were invested on the HER‐2 targeted agents, and significantly improved outcome and prolonged the survival of patients. However, some patients classified as ‘HER‐2‐positive’ would be still resistant to the anti‐HER‐2 therapy. Various mechanisms of drug resistance have been illustrated and the alteration of HER‐2 was considered as a crucial mechanism. However, systematic researches in regard to the HER‐2 mutations and variants are still inadequate. Notably, the alterations of HER‐2 play an important role in drug resistance, but also have a potential association with the cancer risk. In this review, we summarize the possible mutations and focus on HER‐2 variants’ role in breast cancer tumourigenesis. Additionally, the alteration of HER‐2, as a potential mechanism of resistance to trastuzumab, is discussed here. We hope that HER‐2 related activating mutations could potentially offer more therapeutic opportunities to a broader range of patients than previously classified as HER‐2 overexpressed.

## Introduction

HER‐2 is a member of the human epidermal growth factor receptor (HER) family, additionally comprised of epidermal growth factor receptor (EGFR), HER‐3, and HER‐4. These receptors regulate normal cell proliferation, survival, and differentiation *via* different signal transduction pathways 1. The gene encoding HER‐2 is located in chromosome 17, and codes for a 185‐kPa protein that functions as a transmembrane growth factor receptor 2. The intracellular domain of HER‐2 contains approximately 500 residues and composed of three parts: a cytoplasmic juxtamembrane linker, a tyrosine kinase (TK) domain and a carboxyl‐terminal tail 3, 4. The TK domain is more complicated than other parts of HER‐2 receptor, which contains several important loops: the C‐loop (residues 844–845), the αC‐helix (residues 761–775), the N‐loop (residues 727–732) and the activation loop (A‐loop residues 863–884), to form the enzyme active site 3.

Though HER‐2 point or insertion mutations were first described in 2004, researches efforts about them are not exhaustive compared with his family EGFR to date 5. According to the existing data, the probability of HER‐2 mutations is 1.67% in breast cancer, 1–4% in lung cancer and 2.9% in colorectal 6, 7, 8, 9, 10, 11, 12. Other human tumour types have also been reported to harbour HER‐2 mutations, including head and neck cancers, bladder cancers, gastric cancers, ovarian cancers, hepatic cancers 6, 9, 10, 11, 12, 13, 14, 15, 16, 17, 18, 19, 20, 21. Mutational activation of HER‐2 can result from three types of somatic molecular alterations: small insertions and missense mutations in the kinase domain, missense mutations in the extracellular domain, or large deletions of the extracellular domain which yield a truncated form of HER‐2 22, 23. More mutations are mainly located in the three exons (19–21) of the TK domain 24, and are encoded by the DNA sequences in the exons 18–23 25. HER‐2 kinase domain mutations have been described in lung carcinoma and breast cancer albeit at a lower frequency 26, 27, 28, 29. HER‐2 kinase domain mutations can be categorized as: missense point mutations, small in‐frame insertions or duplications which almost occurring in exon 20 and in frame deletions. Among these mutations, the in‐frame insertions or duplications in exon 20 are the most frequently encountered types of mutations 22, 30, 31, 32. In addition, we also take the HER‐2 splice variants into account, including p95HER‐2 and Δ16HER‐2.

The clinical success of gefitinib, an inhibitor of EGFR, in a subset of lung cancers with mutations in the TK domain of EGFR, holds a promise for the future of targeted therapy 33, 34, and also leads to the investigation of analogous mutations of HER‐2. With the application of HER‐2 fluorescent *in situ* hybridization and HER‐2 immunohistochemistry which are standard clinical tests for HER‐2 gene amplification 35, 36, HER‐2 gene amplification or protein overexpression has been extensively studied in breast cancer 37, 38, 39, 40, much less is known about genetic variants and mutations that might have an impact on the risk or therapy of breast cancer. It may be more challenged to successfully target HER‐2 mutations than EGFR mutation. More efforts are needed to translate this idea to clinic.

## The HER‐2 mutations and variants

### The HER‐2 mutations

**Figure 1:**
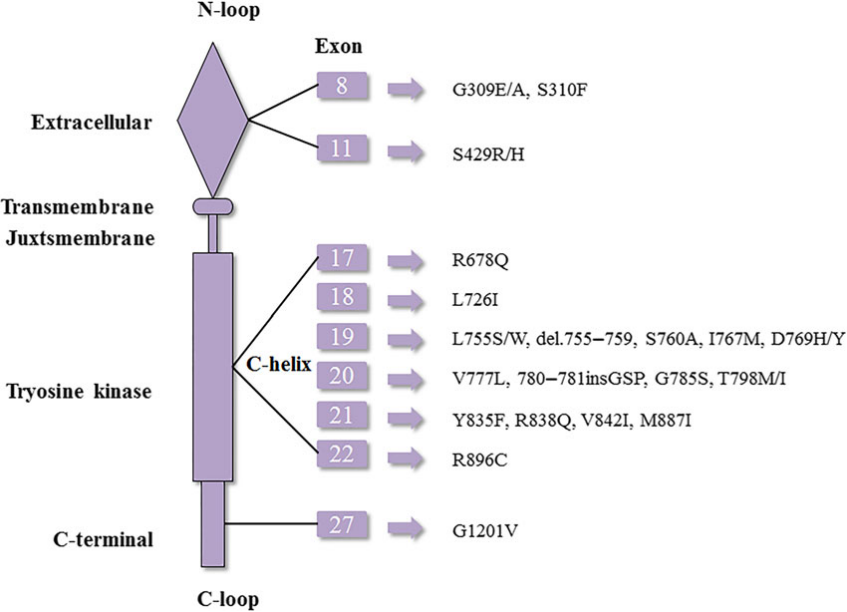
The HER‐2 mutations. These HER‐2 mutations are the common type found in the patients lacking HER‐2 expression and most of them were found in the tyrosine kinase domain. HER: human epidermal growth factor receptor.

These HER‐2 mutations are the common type found in the patients lacking HER‐2 overexpression and most of them were found in the TK domain (Fig. 1).

#### Mutations in TK domain

Human epidermal growth factor receptor‐2 gene amplification or protein overexpression has been identified as a mechanism of HER‐2 activation in breast cancer 1. However, HER‐2 activating mutations, another novel modus to activate HER‐2, have been reported 41, 42. Bose and his colleagues identified 16 HER‐2 somatic mutations though cancer genome sequencing in HER‐2 gene amplification‐negative breast cancer patients. Seven of these HER‐2 kinase domain mutations are activating and oncogenic, including G309A, D769H, D769Y, V777L, P780ins, V842I and R896C 23. Activating HER‐2 kinase domain mutations could also been found at low frequency in several other carcinomas, such as bladder cancer and lung cancer 23, 31, 43.

Human epidermal growth factor receptor‐2 gene with some kinase domain mutations shows the characteristics of constitutively activate kinase activity and increased oncogenicity compared to the wild‐type HER‐2 44. The positive effect of activating mutations on tumour growth has been demonstrated *in vitro* 23. The enhanced kinase activity promotes the formation of the dimmers. Meanwhile, the activating mutations particularly induce the phosphorylation of cellular signalling proteins 23.

The most prevalent activating mutations of HER‐2 involve the insertions within exon 20 and these mutations have stronger catalytic activity 44. These insertions are more potent in transphosphorylating EGFR compared with the wild‐type HER‐2. Conclusively, the mutant HER‐2 gene is more transforming and more capable to inhibit the effect of apoptosis 44.

For the small insertion activating mutations, the basic mechanism is that these mutations lead to a conformational change of the autoinhibition, consequently keeping their inactive condition. The oncogenic insertions frequently alter the Adenosine triphosphate (ATP)‐binding cleft, which forms a conformational structure with many important structures surround the cleft, including phosphate‐binding and activation loops 45.These insertions induce a conformational change of the autoinhibitory αC‐β4 loop, thus, narrowing the ATP‐binding cleft, increasing both the ATP binding affinity and turnover number, and promoting the enhanced kinase activity that participates in the subsequent phosphorylation events 46. In addition, the HER‐2 insertions can potently transphosphorylate EGFR, even in the presence of EGFR tyrosine kinase inhibitors (TKI) 44. It induced transphosphorylation of kinase‐dead EGFR, and exhibited higher ligand‐independent tyrosine phosphorylation. Moreover, the insertions were more potent than wide‐type HER‐2 in associating with signal transducers that mediate proliferative and prosurvival responses 44.The activating missense mutations in kinase domain have also been reported. Most of the mutations were detected in the αC‐helix which is considered to play a critical role in the activation of HER‐2 gene 23. The structures of αC‐helix may be altered by the single missense mutations, and these altered structures might promote tumourigenesis and the phosphorylation of signalling proteins including (phospholipase γ C1, phospholipase Cγ (PLCγ) and mitogen‐activated protein kinase(MAP Kinase)) 25. Some other mutations, such as the HER‐ exon 19 in‐frame deletions 755–759, homologous to EGFR exon 19 in‐frame deletions, could potently increase phosphorylation of EGFR or HER‐3, and interact differently with its dimerization partners compared with other HER‐2 mutants 25. In addition to these activating mutations, some mutations are resistant to the targeted therapy, such as mutations located at codon 755 or 798. The underlying mechanisms of resistance will be discussed in the next sections.

#### HER‐2 mutations in other domain

Apart from the mutations in TK domain, recurring HER‐2 extracellular domain mutations in breast and lung cancer were also identified (*e.g*. S310F/Y, G309A/E, S335C) 23, 47, 48, 49, 50. Parts of these mutations that cluster in exon 8 are oncogenic and activated by two distinct mechanisms, characterized by elevated C‐terminal tail phosphorylation, such as S310F/Y, or covalent dimerization mediated by intermolecular disulfide bond formation, such as G309E and S335C 47. These extracellular domain mutations are also sensitive to small‐molecule inhibitors of HER‐2, more similar to the kinase domain mutations 23. Trastuzumab was shown to be effective against the cells expressing G309 and S310 mutations, giving hope to the patients harbouring these mutations 47.

Recently, novel transmembrane domain mutations were also reported in familial lung adenocarcinoma, including kinds of germline mutations (G660D, V659E and I655V). The V659E was first detected in a case report of Li‐Fraumeni syndrome, and was found to have a oncogenic role 51. G660D and V659E mutations in the transmembrane domain also correlate with the hereditary, sporadic lung adenocarcinomas 52. The G660D and V659E mutations, more stable than wild‐type genes, both can act as driver mutations in lung cancer, and have the capacity to activate Akt. Simultaneously, p38 was also activated to promote cell proliferation in lung adenocarcinoma 52. Additionally, I655V in the transmembrane domain was reported to increase the breast cancer risk.

### The HER‐2 variants

These variants, different from the point mutations, can be defined as incomplete HER‐2 or fragments of HER‐2. They may even appear opposite functions due to their various constructions (Fig. 1).

#### Δ16HER‐2

**Figure 2:**
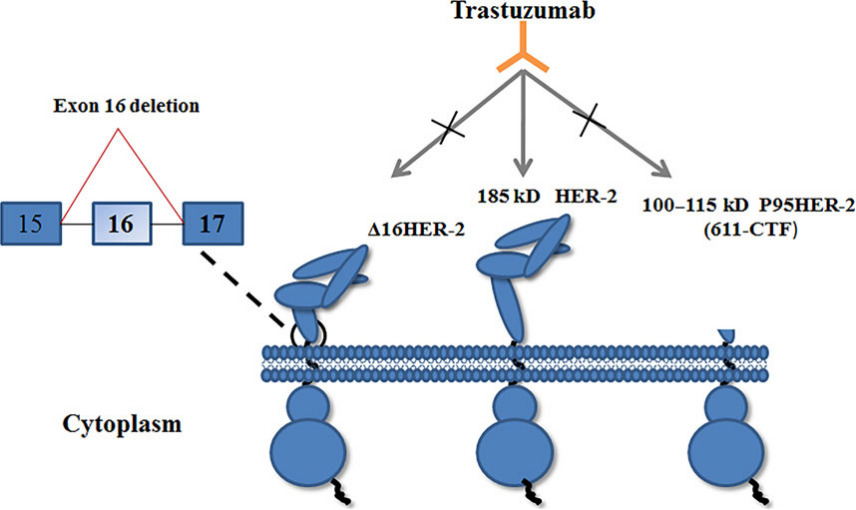
The structure of the ∆16HER‐2 and p95HER‐2 variants. The variants, different from the point mutations, can be defined as incomplete HER‐2 or fragments of HER‐2, including Δ16HER‐2 and p90HER‐2. HER: human epidermal growth factor receptor.

Except for the small insertions or point mutations, some other resistant HER‐2 alterations have also been identified in HER‐2‐positive breast cancer, such as Δ16HER‐2 (a HER‐2 splice variant lacking exon 16) and p95HER‐2 (carboxy‐terminal HER‐2 fragments, mostly known as 611‐CTF; Fig. 2) 53, 54. Both of these alterations could explain the clinical failure of trastuzumab 55, 56, 57, 58, 59, 60. The Δ16HER‐2, a type of oncogenic variant caused by the in‐frame deletion of exon 16 in the extracellular domain of HER‐2, is reported to comprise 4–9% of total HER‐2 transcripts 56, 61. The Δ16HER‐2 variant, when expressed at high levels, harbours enhanced transforming activity compared with wild‐type HER‐2 54. The primary activating mechanism is that this conformation with removed relevant cysteine residues promotes intermolecular disulfide bonding and the generation of homodimers, thus transforming cells 62. Another study found that 44% of Δ16HER‐2‐expressing breast cancer showed the activated Src kinase, heralding the potential clinical implications of direct coupling of Δ16HER‐2 to Src kinase. The activated Src kinase is also associated with the metastatic tumour, suggesting that more aggressive therapeutic interventions are needed 54. ∆16HER‐2 has also been implicated in resistance of HER‐2 positive breast cancers to anti‐HER‐2 therapies. Thus, measurement of this variant may help predict the response to treatment of anti‐HER‐2 therapy. However, to our knowledge, no studies were conducted on the issue till now 63, 64.

#### p95HER‐2

p95HER‐2 is a form of truncated HER‐2, which does not have the complete extracellular domain (Fig. 2). This CTF are yielded through at least two different mechanisms: proteolytic shedding of the extracellular domain of the full‐length HER‐2 receptor or translation of HER‐2 mRNA from alternate internal initiation codons (positions 611 and 678, respectively) 65, 66. Strikingly, *in vitro* studies of 611‐CTF (100–115 kD) revealed more rapid activation of multiple signalling pathways to promote tumour progression when compared with the full length receptor and 648‐CTF 67. p95HER‐2 is hyperactive and has been demonstrated to play a role in cancer progression, increased metastasis, poor prognosis and disease‐free survival when compared with patients that express the wild full‐length HER‐2 65, 68. Approximately 30% of HER‐2‐positive tumours express this HER‐2 fragment 66. The specific characteristic of p95HER‐2 is still unclear, but the overexpression of p95HER‐2 can promote the growth of tumour *via* forming homodimers by intermolecular disulfide bonds in subdomain IV, similar to dimers formed in extracellular domain mutations 69, 70.

Because p95 is thought to be sensitive to HER‐2 active TKI, measurement of quantitative p95 levels may have potential role of treatment decisions in future. Till now, at least two antibodies have been generated against the 611‐CTF form of p95 63, 64. One of the antibodies, D9, has been generated to build a quantitative p95 assay to identify a group of HER‐2 positive patients expressing p95HER‐2 that have a worse outcome while on trastuzumab.

#### Some other variants of HER‐2

Other variants of HER‐2 with contrasting roles in tumour have also been detected, such as Herstatin (results from intron 8 retention) and p100 (results from intron 15 retention) 71. These variants can interfere with the oncogenic activity of wild‐type HER‐2, to inhibit tumour cell growth 56. Further exploration of p100 found a decrease in downstream signal induction. Besides, the protective Herstatin have also been reported to inhibit the activity of HER‐2 by interfering with the phosphorylation of dimmers (HER‐2/HER‐3 and HER‐2/EGFR) 56. Herein, considering of the less association with breast cancer risk or drug resistance, we will not focus on these two subsets of variants more.

## The HER‐2 mutations associated with breast cancer risk

A breadth of literature describes the link between genetic variations and breast cancer risk. Single nucleotide polymorphisms (SNPs) are the commonest sources of human genetic variations that contribute to a susceptibility of tumour progression 72. A polymorphism of the HER‐2 gene that results in the substitution of isoleucine‐to‐valine atcodon 655 of the transmembrane domain (Ile655Val, rs1136201) has been extensively investigated as a risk factor for breast cancer 73. Since the initial case**–**control study on 700 Han chinese women from Xie *et al*. reported a significantly increased risk for carriers of this allele [odds ratio (OR) = 1.4] 74, many epidemiological studies have been conducted to reveal an association between the HER‐2 655V polymorphism and an increased risk of breast cancer 31. However, the results are inconsistent. Several meta‐analyses have been performed to investigate the association between the polymorphism and breast cancer. Due to the differential including studies and methodological issues, different results arise from these meta‐analyses. Lu *et al*. found a significant association among Africans and Asians, but not in Europeans 75. However, Ma *et al*. did not demonstrate any significant associations between HER‐2 codon 655 polymorphism and breast cancer susceptibility, either at the overall or the ethnicity analyses 76. Moreover, a novel updated meta‐analysis suggests that this polymorphism is marginally associated with breast cancer in worldwide populations with additive and dominant models, but not a recessive model 77. Thus, no confirmed associations could be identified between the polymorphism and an increased breast cancer risk among different ethnicities.

A stronger association was revealed in women both under the age of 45 years and with a family history, and the valine allele might not have any effect among women older than 60 78. Additionally, it also raised a possibility that the risk associated with carrying the HER‐2 valine allele might predominantly affect pre‐ or peri‐menopausal breast cancer 78. Consistent with the above investigations, another study also suggested that that V/V or V/I genotype have a twofold increased risk compared with I/I genotype among women who were both younger than 45 years of age and reported a positive family history of breast cancer (OR = 2.3, 95% CI = 1–5.3) 79. However, some conflicting results emerged, revealing that HER‐2 I655V polymorphism may be a biomarker for breast cancer susceptibility among older women 80. Furthermore, a rare HER‐2 variant Ile654Val is also associated with an increased familial breast cancer risk, which revealed an oncogenic role for carriers of the heterozygous Val654 allele (OR = 2.56, 95% CI = 1.08–6.08, *P* = 0.028) 81, meanwhile, it is linked with the more frequent Val655 to form two consecutive valines instead of two isoleucine residues 81.

**Figure 3:**
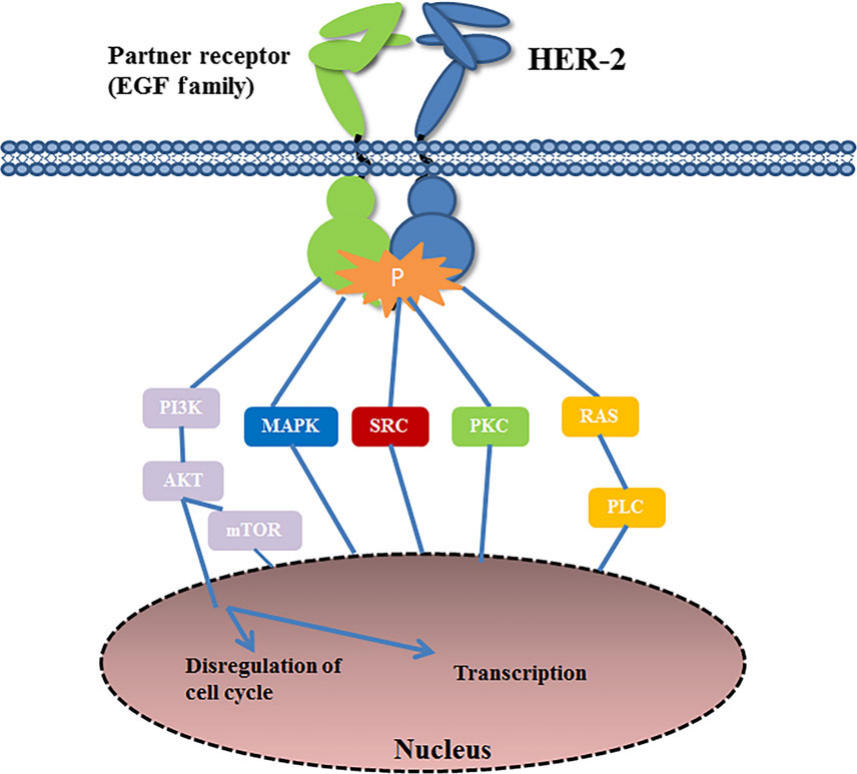
Working model for the human epidermal growth factor receptor (HER)‐2 oncogenic activities in breast cancer development and progression.

Human epidermal growth factor receptor‐2 is considered an orphan receptor since it is the only receptor of HER family in the absence of an identified ligand (Fig. 3) 82, 83. However, HER‐2‐containing heterodimers could function as the most active signalling complex of the HER family 84, 85. A strong pro‐tumourigenic signalling cascade is initiated by the overexpression of HER‐2, and leads to the generation of dimmers 86, 87. A subsequent activation of HER‐2 cytoplasmic kinase activity is needed for the downstream PI3K/AKT signalling pathways to promote cell proliferation and the effect of apoptosis (Fig. 3) 85, 87, 88. The basic activating mechanism of HER‐2 variants is to enhance the kinase activity and cell transformation by increasing the formation of active HER‐2 heterodimmers 72. The implication of HER‐2 polymorphism in tumour progression may preferentially occur through the modification of function rather than the amplification of protein 72. Furthermore, the independent genetic variant in growth factors signalling appear to have the stronger influence on breast cancer risk *via* combining with other variant gene, including variant fibroblast growth factor 1 (FGF1), FGF2 and neuregulin 2 (NRG2), interacted with SNPs in platelet‐derived growth factor B (PDGFB), EGFR, HER‐2 and FGFR2 89.

## The HER‐2 mutations associated with breast cancer resistance

In 1987, HER‐2 amplification and overexpression were first reported. These molecular alterations could be found in approximately 20–30% of breast cancer 90. Since then, HER‐2 was considered as a significant targeted point due to its distinctive role in tumour cell proliferation and metastasis, facilitating the development of HER‐2 targeted agents, which have shown a tremendous success 91. However, the efficacy of anti‐HER‐2 therapeutics such as trastuzumab or small molecule HER‐2 TKI (lapatinib) is limited by the occurrence of therapeutic resistance 92, 93. Major mechanisms of primary or acquired resistance against these targeted agents include 94: (*i*) Alteration in binding sites or TK receptor domain. (*ii*) Up‐regulation of alternative ErbB ligands and dimerization of receptors to counteract for receptor inhibition. (*iii*) Dimerization/interaction with other receptors. (*iv*) Downstream controllers‐deficient tumours. (*v*) Activation of downstream signalling and survival pathways. Here we mainly discuss the alteration of HER‐2 gene (Table 1).

**Table 1:** The HER‐2 mutations and variations associated with breast cancer resistance

Mutations	Primary tumour	Number of patients screened	TNM stage	Drug resistance	Reference
L726I	Cell study	NA	NA	Gefitinib	104
L726F	NA	76	NA	Lapatinib	42
L726F	Cell study	NA	NA	Lapatinib	101
L755S	Invasive ductal	94	IIIA	Lapatinib	7
L755S	Invasive ductal	94	IIA	Lapatinib	7
L755S	Lobular	193	NA	Lapatinib	28
L755S	TNBC	104	NA	Lapatinib	29
L755S	Lobular	1499	IIA	Lapatinib	23
L755S	Ductal	1499	I	Lapatinib	23
L755P	Cell study	NA	NA	Lapatinib	103
P780L	Cell study	NA	NA	Lapatinib	101
S783P	Cell study	NA	NA	Lapatinib	101
L785F	Cell study	NA	NA	Lapatinib	101
T798M	Cell study	NA	NA	Lapatinib/Trastuzumab	97
T798M	Cell study	NA	NA	Lapatinib	103
T798I	Cell study	NA	NA	Lapatinib	101
T798I	Cell study	NA	NA	Lapatinib	102
p95HER‐2	NA	483	NA	Trastuzumab	68
Δ16HER‐2	NA	NA	NA	Trastuzumab	62

NA: not available; TNBC: triple negative breast cancer; HER: human epidermal growth factor receptor.

### The point or small insertion resistance mutations

To our knowledge, HER‐2 point or small insertions were found predominantly in patients lacking HER‐2 amplification to date 24. It suggests that the mutant gene may not be associated with real amplification 95. In fact, insufficient evidence about the mutations in HER‐2 ‘positive’ can be searched. Among these limited researches, rare patients harbouring HER‐2 amplification were tested for the presence of mutations. Hence, it is an arduous work to obtain any conclusions about relative characteristics. Herein, we conclude some possible reasons that could explain this low frequency event, including: one possible reason is that these mutations may be acquired only after the utilization of anti‐HER‐2 agents, suggesting that the anti‐HER‐2 agents might be a trigger to the generation of resistance alterations. Another possible explanation is that these mutations only occupy small parts of the amplified HER‐2, thereby, may not be detected by the DNA sequencing methods due to below the limits of sensitivity 95. Further investigations are needed to clarify the specific mechanisms.

These unusual intrinsic mutations in HER‐2 overexpressed patients occur with an inconspicuous probability. It is reported that a 52‐year‐old man diagnosed with stage IV non‐small cell lung cancer (NSCLC), was detected to overexpress HER‐2 and harbour an L869R mutation. Later he achieved a partial response to lapatinib, but showed no response to trastuzumab alone. The mutation in this patient is analogue to the previously reported L861 mutation in EGFR 7, 96. But whether the resistance is caused by the mutation or not is still unknown. Another study revealed that the HER‐2 gene‐amplified breast cancer cells, which harbour the T798M mutant alleles, acquired a resistance to both lapatinib and trastuzumab alone. However, after the treatment of a simultaneous blockade of HER‐2 and EGFR, an effective response was shown, hinting a possible connection between increased EGFR ligand production and drug resistance 97. Additionally, it should be noted that T798 is a gatekeeper residue, analogous to the gatekeeper EGFR T790M 98, ABLT315I 99 and cKITT670I 100 mutations 97, which are all related to the clinical drug resistance. All above results indicate that the amino acids L755 and T798 in HER‐2 are critical residues, enable to determine lapatinib sensitivity. Strong lapatinib resistance caused by L755S, L755P and T798M, T798I have been reported 101, 102. The frequency of T798 mutations is higher than other mutations 23, 103. Despite the resistance mutation, activating mutation (D769H) have also been detected in HER‐2 positive patients. But whether some associations exist between activating mutations and HER‐2‐positive patients is unclear yet 23.

Apart from the above conditions, the mutations could also occur after the treatment of anti‐agents. The wild‐type HER‐2 could acquire the L755S and T862A mutation after the exposure of lapatinib, suggesting that kinase domain mutations may cause a secondary resistance in patients with wild type HER‐2 103. Bose and his colleagues identified that L755S mutation was the most common subtype of mutant HER‐2 in breast cancer. In his study, six of total 27 patients with mutant HER‐2 were detected with the L755S mutation 23. Similar condition occurred after the treatment of other agents, such as gefitinib or iressa, a selective epidermal growth factor receptor TKI, primarily for NSCLC, also respond to breast cancer with positive HER‐2. A subset of breast cancer cell lines overexpressing the activated HER‐2 was treated with 5 Mmol/l gefitinibt. Then a novel point mutation‐L726I in the ATP‐binding pocket was found, enabling these cells insensitive to gefitinib 104.

Most HER‐2 mutations associated with lapatinib resistance locate in the ATP‐binding and hinge region. Herein, we mainly discuss the mechanism of L755 and T798 due to their vital roles. Mutations at L755 can conclusively stabilize the active conformation of the HER‐2 kinase. They may not directly affect inhibitor binding, but form a conformation where the αC‐helix is fixed in to inhibit lapatinib binding and influence the structure of the active state 103. As the HER‐2 ‘gatekeeper’, T798 is located in exon 20 within hinge region, which is the most prominent site of resistance mutations. Mutations at T798 may lead to the obstacle of TKI by directly interfering with the steric structure 101. One mechanism is to increase the affinity of HER‐2‐T798M towards ATP, similar to the T790M mutation in EGFR. Another mechanism is that lapatinib binds the inactive conformation preferentially, then incapable to bind the active conformation in T798M 103. Additionally, another studies revealed that cells harbouring T798M mutation showed increased expressions of the EGFR ligands EGF, transforming growth factor‐α, amphiregulin and proheparin‐binding epidermal growth factor (HB‐EGF), leading to the resistance of TKI 97. Besides, the ability of HER‐2YVMA's mutation to amplify their transforming potential and modify tumour microenvironment through induction of growth factors was recently demonstrated 105.

### The resistance variants of HER‐2

Human epidermal growth factor receptor‐2 variants were identified in patients with HER‐2 amplification, and conferred resistance to targeted therapy. These variants mainly include Δ16HER‐2 and p95HER‐2.

The patients harbouring this Δ16HER‐2 are also refractory to the treatment of trastuzumab. The mechanism of this clinical failure still needs exploration. The potential oncogenic properties were mediated through a direct interaction between Δ16HER‐2 and Src kinase. Treatment with single‐agent TKI dasatinib overcame the resistance to trastuzumab, and suppressed tumourigenicity. In addition, the capacity of stabilizing HER‐2 homodimers and the phosphorylated state of phosphatase and tensin homolog (PTEN) may also contribute to the resistance 54. Surprisingly, trastuzumab was even identified to promote the growth and invasion of tumour cell 54. Interestingly, it also demonstrated that 89% of patients with the expressed Δ16HER‐2 were locally disseminated node‐positive breast cancer, indicating that more attention should be put on this subtype 56.

p95HER‐2 is hyperactive, and was described as a truncated form of HER‐2 lacking the antibody's binding region. It has been demonstrated to be associated with cancer progression, metastasis, poor prognosis and disease‐free survival when compared with patients expressing the wild full‐length HER‐2 65, 68. Additionally, patients with breast cancer harbouring the expression of p95HER‐2 exhibit less response to trastuzumab compared to patients without p95HER‐2. The lack of outer‐cell attachment domain containing the binding site for trastuzumab results in the failure of drug binding. Besides, actively signalling protein generated by ectopic expression of p95HER‐2, also promotes trastuzumab resistance that has been demonstrated in preclinical and clinical studies 95.

## The therapy methods to these HER‐2 mutations

Recently, more researches about the HER‐2 mutations have been investigated. Most of the mutations were described in patients without HER‐2 overexpression or amplification. These have brought us a profound change to the traditional targeted therapy for patients with mutant HER‐2. Studies about HER‐2 mutations, both *in vitro* and *in vivo*, have proved that the applicability of anti‐HER‐2 agents, such as inhibitor combinations, lapatinib plus trastuzumab, or afatinib plus rapamycin, are the most effective therapy in HER‐2‐mutant cancers 106, 107. Patients with HER‐2‐mutant NSCLC were also reported to have achieved disease control with anti‐HER‐2 therapy 108, 109. Similar outcome of HER‐2‐targeted therapy has also been achieved in breast cancer patients with HER‐2‐mutantions 103, 110. Notably, in Bose's research, all HER‐2 mutations including mutation L755S which is resistant to lapatinib, exhibited sensitivity to the irreversible HER‐2 inhibitor, neratinib 23. All these findings validate that patients with HER‐2‐mutant could benefit from existing HER‐2 targeted therapy, particularly irreversible inhibitors, such as neratinib. More preclinical and clinical trials should be designed to investigate the application of HER‐2 targeted therapy in HER‐2 mutation positive patients. To date, a prospective, multicenter clinical trial is being launched to screen patients with metastatic breast cancer for HER‐2 mutation and investigate the clinical outcome of HER‐2 targeted therapy (NCT01670877).

Previous researches have revealed that tumours contain p95HER‐2 are resistant to trastuzumab, but still sensitive to the TKI, such as lapatinib 53. In addition, some studies also reported that these TKIs could also provide an effective solution for the patients expressing mutant Δ16HER‐2, suggesting that the TKI may be an alternative therapy for HER‐2 mutations 56. Furthermore, another study indicated that p95HER‐2 could be a possible biomarker to evaluate the efficacy of therapeutic regimens including lapatinib and chemotherapy, and overcome the clinical failure of trastuzumab monotherapy. The effect of lapatinib, as single agents or in combination with other drugs, may be equal in patients regardless of the p95HER‐2 expression 70. Interestingly, a study revealed that tumours expressing these p95HER‐2 fragments, also respond to trastuzumab plus chemotherapy, advocating that p95HER‐2 is also a predictive biomarker for the patients treated with trastuzumab and chemotherapy 53. However, no inhibitors were directly targeted against p95HER‐2. To improve the outcome of patients harbouring these two variants, new researches are still on the way.

## Conclusion

During the past decades, the application of trastuzumab, which targeted against HER‐2, has significantly improved the outcome and prognosis of HER‐2‐overexpressing breast cancer. However, despite the clinical success, the resistance of HER‐2 targeted agents occurred. Primary activating mutations and acquired secondary mutations were detected to play a critical role in breast cancer progression and drug resistance, revealing a sophisticated challenge for the effective treatment of HER‐2 targeted therapy. Lots of preclinical data suggest that the combination of multi‐points HER‐2 targeted therapy may break the drug resistance. This will require an ‘individualized diagnosis and treatment’ based on the detailed molecular analysis of tumours both before and after progression on primary HER‐2 targeted therapy. Future works are still needed to evaluate the role of altered HER‐2 as future prognostic and predictive factors, as well as potential therapeutic targets, and providing ‘an individualized strategy’ for patients with HER‐2 mutant.

## Conflicts of interest

The authors do not have any financial interests to publish this article.
